# Effect of Teriflunomide and Dimethyl Fumarate on Cortical Atrophy and Leptomeningeal Inflammation in Multiple Sclerosis: A Retrospective, Observational, Case-Control Pilot Study

**DOI:** 10.3390/jcm8030344

**Published:** 2019-03-12

**Authors:** Robert Zivadinov, Niels Bergsland, Ellen Carl, Deepa P. Ramasamy, Jesper Hagemeier, Michael G. Dwyer, Alexis A. Lizarraga, Channa Kolb, David Hojnacki, Bianca Weinstock-Guttman

**Affiliations:** 1 Buffalo Neuroimaging Analysis Center, Department of Neurology, Jacobs School of Medicine and Biomedical Sciences, University at Buffalo, State University of New York, Buffalo, NY 14203, USA; npbergsland@bnac.net (N.B.); ecarl@bnac.net (E.C.); dramasamy@bnac.net (D.P.R.); jhagemeier@bnac.net (J.H.); mgdwyer@bnac.net (M.G.D.); 2 Center for Biomedical Imaging at the Clinical Translational Science Institute, University at Buffalo, State University of New York, Buffalo, NY 14203, USA; 3 Jacobs Multiple Sclerosis Center, Department of Neurology, Jacobs School of Medicine and Biomedical Sciences, University at Buffalo, State University of New York, Buffalo, NY 14203, USA; aalizarr@buffalo.edu (A.A.L.); cmkolb@buffalo.edu (C.K.); hojnacki@buffalo.edu (D.H.); bw8@buffalo.edu (B.W.-G.)

**Keywords:** multiple sclerosis, teriflunomide, dimethyl fumarate, cortical atrophy, brain atrophy, leptomeningeal enhancement

## Abstract

**Background:** Pathologic changes in cortical gray matter (GM) and leptomeninges contribute to disability worsening in patients with multiple sclerosis (MS), but there is little evidence whether disease-modifying treatments can slow down cortical pathology in MS. **Objectives:** To investigate the effect of teriflunomide (TFM) and dimethyl fumarate (DMF) in reducing cortical pathology, as determined by percentage cortical volume change (PCVC) and leptomeningeal contrast enhancement (LMCE) on MRI. **Methods:** This was a retrospective, single-center, observational study that selected 60 TFM- and 60 DMF-treated MS patients over 24 months. **Results:** TFM had a lower rate of PCVC compared to DMF over 24 months (−0.2% vs. −2.94%, *p* = 0.004). Similar results were observed for percentage GM volume change over 0–12 (*p* = 0.044) and 0–24 (−0.44% vs. −3.12%, *p* = 0.015) months. No significant differences were found between the TFM and DMF groups in the frequency and number of LMCE foci over the follow-up. TFM showed a numerically lower rate of whole brain atrophy over 24 months (*p* = 0.077), compared to DMF. No significant clinical or MRI lesion differences between TFM and DMF were detected over follow-up. **Conclusions:** These findings suggest that TFM has a superior effect on the preservation of cortical GM volume, compared to DMF.

## 1. Introduction

Cortical gray matter (GM) pathology in multiple sclerosis (MS) is characterized by the presence of cortical subpial lesions [1] and leptomeningeal (LM) inflammation [2,3]. Inflammatory cells in the leptomeninges may act to sustain the immune response, contributing to development of subpial cortical lesions [3]. Gadolinium (Gd)-based three-dimensional fluid-attenuated-inversion recovery (3D-FLAIR) MRI contrast enhancement (CE) shows that LM inflammation occurs relatively frequently in MS patients in vivo [2,4,5], and may be associated with subpial cortical demyelination on post-mortem examination [2].

Focal subpial cortical lesions are related to the development of cortical GM atrophy, as has been demonstrated by neuropathologic evidence implicating significant neuro-axonal and synaptic loss in both early and late stages of the disease [1]. The clinical validity of the assessments of cortical atrophy and leptomeningeal contrast enhancement (LMCE) has been demonstrated in a number of recent short-, mid-, and long-term studies [2,4,6,7,8,9].

There has been lately an increasing interest in understanding the effects of disease-modifying therapies (DMTs), for the treatment of relapsing MS, on cortical GM pathology [8,10,11,12,13,14]. However, no studies have examined the effect of DMTs on cortical atrophy and LMCE longitudinally. In addition, head-to-head clinical trials of different DMTs on cortical pathology have not been conducted.

Teriflunomide (TFM) is an oral immunomodulator that selectively and reversibly inhibits dihydroorotate dehydrogenase, a mitochondrial enzyme essential for de novo pyrimidine synthesis [15]. In a preclinical model (Theiler’s murine encephalitis virus), TFM modulated glutamatergic dysregulation and microglial density in the cortex-basal ganglia-thalamus circuit, thereby reducing possible excitotoxicity, inflammation, and axonal damage [16,17]. In randomized, double-blind, placebo-controlled phase 3 studies in patients with relapsing MS, TEMSO (NCT00134563) [18] and TOWER (NCT00751881) [19], and in patients with clinically isolated syndrome (CIS), TOPIC (NCT00622700) [20], TFM demonstrated significant efficacy over two years in the reduction of clinical and MRI lesion activity measures of disease activity, and whole brain atrophy [21,22], compared to placebo.

Dimethyl fumarate (DMF) is an oral agent which provides anti-oxidative and anti-inflammatory effects through the activation of the nuclear factor erythroid-2 related factor 2 (Nrf2) [23]. In randomized, double-blind, placebo-controlled phase 3 studies in patients with relapsing MS, DEFINE (NCT00420212) [24] and CONFIRM (NCT00451451) [25], DMF demonstrated significant efficacy over two years in the reduction of clinical and MRI lesion measures of disease activity, compared to placebo. Furthermore, DMF slowed down the rate of brain atrophy in DEFINE [26], but not in the CONFIRM study [27].

In a recently completed head-to-head, retrospective, multi-center, longitudinal, clinical, and MRI comparator study between TFM and DMF, Teri-RADAR, TFM showed a significantly lower annualized rate of whole brain volume loss, compared to DMF over 15 months [28].

Against this background, we aimed to extend previous observations from the Teri-RADAR study [28], and investigate the effect of TFM in reducing cortical GM atrophy and LM inflammation over 24 months, as compared to DMF.

## 2. Methods

### 2.1. Study Design and Subjects

This was a retrospective, single-center, observational, longitudinal (24 months), single-blinded MRI study selecting 120 MS patients, who obtained their routine clinical and MRI examinations after starting treatment with TFM (*n* = 60) or DMF (*n* = 60).

Inclusion criteria were: (1) patient diagnosed with MS according to McDonald criteria [29], (2) age 18–65, (3) relapsing disease course, (4) Expanded Disability Status Scale (EDSS) ≤5.5, (5) MRI obtained on 1.5T or 3T using standardized MRI protocol, (6) patients treated with 14 mg of TFM once daily or DMF 240 mg twice daily for at least three consecutive months, and (7) obtaining MRI within a 30 day window prior to staring the DMT. Exclusion criteria consisted of diagnosis of non-relapsing MS, use of experimental drug or investigational procedure during the study period, pregnancy, severe hepatic impairment, relapse or corticosteroid use within 30 days prior to baseline MRI scan, and previous use of leflunomide, alemtuzumab, cladribine, rituximab, or mitoxantrone. Patients were not eligible for the study if they had previously been treated with TFM or DMF, or if they switched to either of these DMTs during the study.

In order to reduce the potential for selection bias, patients receiving TFM or DMF were selected sequentially in matched pairs from all available cases who met the inclusion and exclusion criteria in our center, until 60 patients in each treatment arm were assigned. Once an eligible treatment pair was identified, the pair was selected into the study. The matching (ratio 1:1) was performed in a way that a TFM patient was identified first, then searched for a matching DMF patient who was within ±3 years of age, ±2 years of MS diagnosis year, and ±0.5 points of EDSS score. The patients were screened from a cohort of over 500 patients treated with TFM or DMF at our center in the period 2012–2016. Of the 124 patients who started treatment with TFM in our center in the period 2012–2016, the mean age was 49.2 (8.4) years, the mean disease duration was 13.8 (7.5) years, the median EDSS was 2.9 (interquartile range 2.5–3.5), and the mean annual relapse rate in 24 months prior to starting the treatment was 0.4 (0.6). There were no significant differences for any of the demographic or clinical characteristics of the TFM patients who were or were not selected in the study. Of the 398 patients who started treatment with DMF in our center in the period 2012–2016, the mean age was 44.3 (10.1) years, the mean disease duration was 11.8 (8.2) years, the median EDSS was 2.5 (interquartile range 2.0–3.5), and the mean annual relapse rate in 24 months prior to starting the treatment was 0.23 (0.5).

Anonymized patient data including demographics, disability (EDSS) scores, and relapses with associated hospitalizations, serious adverse events, or corticosteroid use were collected retrospectively from electronic medical charts, and MRI scans were obtained from the local Picture Archiving and Communication Systems (PACS).

The study and collection of the retrospective data were approved by the local Institutional Review Board (IRB).

### 2.2. MRI Acquisition and Analyses

Baseline and follow-up scans were identified on the same 3T or 1.5T GE Signa Excite HD 12.0 (General Electric, Milwaukee, WI, USA) scanners, using a multi-channel head and neck (HDNV) coil. At baseline, 39 TFM- and 48 DMF-treated patients had their MRI scan on the 3T scanner, whereas 21 TFM and 12 DMF patients were examined on the 1.5T scanner (*p* = 0.101).

The 3T and 1.5T sequences at baseline, 12 months, and 24 months were acquired with a matrix of 256 × 192 and a field of view (FOV) of 25.6 cm with 75 percent phase FOV. The in-plane resolution was 1 × 1 × 3 mm^3^ without gap for 2D T2-fluid-attenuated inversion recovery (FLAIR), and 1 × 1 × 1 mm^3^ without gap for 3D T1-weighted images (WI). Additional sequence parameters for the 3T T2-FLAIR included the following: echo time (TE)/inversion time (TI)/repetition time (TR) = 120 ms/2100 ms/8500 ms. The additional parameters for the 1.5T T2-FLAIR were as follows: TE/TI/TR = 120 ms/2000 ms/8000 ms. The 3D high resolution T1-WI used a fast, spoiled, gradient echo with magnetization-prepared inversion recovery pulse, and the parameters were TE/TI/TR = 2.8/900/5.9 ms, flip angle (FLIP) = 10° for 3T and TE/TI/TR = 3.7/900/7.7 ms, FLIP = 10° for 1.5T. In addition, we acquired 2D T1-WI spin-echo sequence without and 5 min after 0.1 mMol/Kg gadolinium (Gd)-diethylenetriamine penta-acetic acid (DTPA) injection. The parameters were TE/TR = 16/600 ms for 3T, and TE/TR = 12/450 ms for 1.5T.

The presence and frequency of LMCE was assessed at baseline, 12 months, and 24 months only using a post-3D-FLAIR sequence on a 3T scanner acquired 10 min after Gd injection in a sagittal acquisition with a TR/TI/TE = 9000 ms/2420 ms/600 ms, acquisition matrix 256 × 192, voxel size = 1.0 × 1.3 × 1.3 mm^3^, frequency direction inferior to superior. The sequence used variable flip angles to increase the T2 relaxation time [30]. Post-contrast 3D-FLAIR images were present in 39 TFM- and 48 DMF-treated patients at baseline, 35 TFM- and 47 DMF-treated patients at 12 months, and 30 TFM- and 40 DMF-treated patients at 24 months.

Because we implemented identical pre-contrast 3D–FLAIR sequence only in 2015, 29 TFM- and 36 DMF-treated patients at baseline, 26 TFM- and 35 DMF-treated patients at 12 months, and 23 TFM- and 30 DMF-treated patients at 24 months received pre/post-contrast 3D-FLAIR images.

### 2.3. MRI Analyses

All MRI analyses were blinded to the clinical and treatment status of the study participants.

#### 2.3.1. Brain Volume Analyses

For baseline analyses, FMRIB’s SIENAX software was used (version 2.6, FMRIB, Oxford, UK) on 3D T1-WI [31]. Corrections for T1-hypointensity misclassification were performed by lesion filling the images prior to segmentation [32]. Normalized volumes of whole brain, GM, white matter (WM), cortical volume (CV), and lateral ventricle volume (LVV) were measured [31]. In addition, normalized volumes of the thalamus and deep GM (DGM) at baseline were calculated using FIRST (version 1.2, FMRIB, Oxford, UK) [33].

For longitudinal changes of the whole brain volume, we applied the SIENA method to calculate the percentage brain volume change (PBVC) [31]. To quantify longitudinal GM (PGMVC), WM (PWMVC), and cortical (PCVC) percentage volume changes, we used SIENAX-multi timepoint (SX-MTP) (version 1.0, Buffalo Neuroimaging Analysis Center, Buffalo, NY, USA) [34]. The percentage LVV change (PLVVC) was evaluated using VIENA (version 1.0, VU medical center Amsterdam, The Netherlands) [35]. To quantify longitudinal volume change of the thalamus and DGM, we used FIRST [33].

#### 2.3.2. LMCE Detection

LMCE foci were defined as signal intensity within the subarachnoid space that was substantially greater than that of brain parenchyma on post-contrast 3D-FLAIR scans, as reported previously [4,36]. The images were reviewed, using JIM (version 6.0, Xinapse Systems, Northants, UK) software (www.xinapse.com), in the sagittal plane of the original acquisition and in additional views. LM inflammation was evaluated according to the presence and number of LMCE foci.

The detection of LMCE foci was performed separately on post-contrast 3D-FLAIR images in native space for all subjects who were scanned on 3T, and on a subset of subjects who obtained pre/post-contrast coregistered and subtracted 3D-FLAIR images, as previously described [36]. The two analyses were performed six months apart (first the post-contrast and then the pre/post-contrast) and by different blinded readers. Briefly, the 3D FLAIR post-contrast image was rigidly registered to the 3D FLAIR pre-contrast image [31], and the 3D FLAIR pre-contrast image was then voxel-wise subtracted from the coregistered 3D FLAIR post-contrast image, yielding a subtraction map, which was subsequently smoothed with a Gaussian kernel of 0.5 mm.

#### 2.3.3. Lesion Activity

Gd+ and new/enlarging T2 lesion number and volume were measured on post-contrast T1 and FLAIR images, respectively. T1-hypointense lesions were measured on pre-contrast T1 images.

### 2.4. Endpoints

The primary outcome was to determine the effect on PCVC between TFM and DMF over 24 months.

The secondary endpoint was to determine the frequency of LM inflammation, as measured by the presence and number of LMCE foci, between TFM and the DMF, over 24 months.

Post-hoc analyses included evaluating differences on clinical and MRI lesion measures of disease activity between the treatment arms.

### 2.5. Statistical Analysis

Analyses were performed using the Statistical Package for Social Science (SPSS), 24.0 (IBM, Armonk, NY, USA).

A priori sample size determination for this study was based on the calculation of PBVC differences over 15 months from the recent Teri-RADAR study [28], where the following figures were observed: −0.2% (SD 0.83) for the TFM group versus −0.71% (SD 1.1) for the DMF group, yielding an effect size of *d* = 0.53. Considering PBVC as a proxy for cortical GM volume loss (PCVC), and an effect size of *d* = 0.53, a minimum sample of 57 patients per group was needed to achieve 80% power. In order to account for possible treatment cessations, we selected 60 patients in each treatment arm for a total of 120 patients.

The primary analysis of each endpoint was based on observed data (i.e., no data imputation was performed for missing data).

Differences between groups were analyzed using chi-squared (χ^2^) test, Student’s *t*-test, and the Mann–Whitney rank-sum test, as appropriate. MRI differences between the treatment arms were calculated using analysis of covariance (ANCOVA), corrected for age and sex.

A nominal *p*-value of ≤0.05 was considered statistically significant, using two-tailed tests.

## 3. Results

### 3.1. Demographic, Clinical, and MRI Differences at Baseline between the Two Treatment Groups

Table 1 shows demographic and clinical characteristics at baseline of the 120 relapsing MS patients who met the inclusion criteria and started treatment with TFM (*n* = 60) or DMF (*n* = 60). There were no differences between the groups in age (*p* = 0.879), age at onset (*p* = 0.869), sex (*p* = 0.820), disease duration (*p* = 0.674), relapse rate in previous 12 (*p* = 0.351) or 24 (*p* = 0.779) months, or in the EDSS score (*p* = 0.377). The type of DMT in the previous 12 months was similarly distributed between the two treatment arms (*p* = 0.144).

Table 2 shows baseline lesion and brain volumetric characteristics of the 120 relapsing MS patients who started treatment with TFM or DMF. No significant differences were found between the two treatment groups for cortical volume (*p* = 0.573) or other brain volume measures. Only 4 (10.3%) of TFM and 8 (16.7%) of DMF patients presented with LMCE at baseline (*p* = 0.389), and there were no differences between the two groups in the number of foci (*p* = 0.718). Similar LMCE frequency figures were observed for pre/post-contrast 3D-FLAIR coregistered and subtracted images for TMF (*n* = 4) and DMF (*n* = 7) patients. Finally, 16.7% of the TFM and 21.7% of the DMF patients were Gd-positive at baseline (*p* = 0.626).

### 3.2. Clinical Characteristics between the Two Treatment Groups over the Follow-Up

Table 3 shows clinical characteristics of the TFM- and DMF-treated patients over the follow-up. There were no significant differences between the two treatment groups in the time of follow-up (25.8 vs. 27.9 months, *p* = 0.564). Of 60 TFM-treated patients who were selected in the study, all completed their 12-month follow-up, however 12 TFM-treated patients stopped the treatment between 12 and 24 months. Of 60 DMF-treated patients, 3 stopped the treatment in the first 12 months, and an additional 11 between the 12 and 24 months. The cessation rate was not significantly different between the two treatment groups (*p* = 0.214). A similar number of TFM- and DMF-treated patients started interferon beta-1a, glatiramer acetate, or natalizumab after discontinuation of either TFM or DMF (*p* = 0.950). No serious adverse events were reported. The main reasons for switch of the DMT in both TFM and DMF groups were occurrence of relapses or perception of lack of efficacy.

No significant differences in relapse rate between the 0–12 (*p* = 0.683), 12–24 (*p* = 0.412), or annualized relapse rate between 0–24 (*p* = 0.890) months were observed between the two treatment groups. In total, there were 19 relapses in 18 TFM-treated patients, and 22 relapses in 22 DMF-treated patients over the follow-up (Table 3). There were also no significant differences between the two treatment arms in EDSS at 12 (*p* = 0.377) or 24 (*p* = 0.339) months, or EDSS absolute change between 0–12 (*p* = 0.342), 12–24 (*p* = 0.626), or 0–24 (*p* = 0.722) months. No patients transitioned to secondary-progressive MS during the study.

### 3.3. Differences in Percentage Brain Volume Changes between the Two Treatment Groups

Table 4 shows that TFM-treated patients had a lower rate of PCVC compared to the DMF-treated group over 24 months (−0.2% vs. −2.94%, *p* = 0.004). Although TFM-treated patients had a lower rate of PCVC over 0–12 (−0.29% vs. −1.48%) and 12–24 (−0.79% vs. −1.6%) months compared to the DMF-treated patients, these differences were not significant. Similar PCVC findings over 24 months were observed when only patients with 3T examinations were analyzed (*p* = 0.01; Table S1).

A post-hoc analysis (Table 4) showed that TFM-treated patients had a lower rate of PGMVC over 0–12 (−0.24% vs. −1.71%, *p* = 0.044) and 0–24 (−0.44% vs. −3.12%, *p* = 0.015) months, compared to DMF-treated patients. Similar PGMVC findings over 24 months were observed in the 3T subcohort between the TFM and DMF patients (*p* = 0.001, Table S1). However, no statistically significant differences between the two treatment groups were observed for thalamus (PTVC) and DGM (PDGMVC) percentage volume changes over any time point of the study for the entire cohort of patients (Table 4). However, the 3T TFM subcohort showed significantly lower PTVC (*p* = 0.046) and PDGMVC (*p* = 0.008) over 24 months compared to the DMF one (Table S1). Post-hoc analysis showed there was a numerically lower rate of PBVC in TFM- compared to DMF-treated patients over 0–24 months (−1.45% vs. −2.07%, *p* = 0.077), which was also numerically different for the 3T subcohort, but not significantly so (*p* = 0.188). There was also significantly lower PLVVC in the TFM- compared to the DMF-treated group over the first 12 months of the study (1.40% vs. 3.56%, *p* = 0.05), but while the difference was numerically different (3.85% vs. 6.26%) over 24 months, it was not significant (*p* = 0.245).

### 3.4. Differences in the Frequency and Number of Leptomeningeal Contrast Enhancement Foci between the Two Treatment Groups

Table 5 shows no significant differences in frequency and number of LMCE foci between the two treatment groups over the follow-up for post-contrast 3D-FLAIR images analyzed in native space for all subjects scanned on 3T MRI, and for a subset of subjects who obtained pre/post-contrast coregistered and subtracted images at all time points of the study.

On post-contrast 3D-FLAIR images analyzed in native space, of 8 DMF-treated patients who presented with LMCE foci at baseline, 6 continued to show the same LMCE foci at 12 and 24 months, whereas one new patient treated with DMF developed a new LMCE focus over the follow-up. Of 4 TFM-treated patients who presented with LMCE foci at baseline, 2 patients continued to present the same LMCE foci at 12 and 24 months, whereas one patient treated with TFM developed a new LMCE focus over the follow-up.

On pre/post-contrast 3D-FLAIR coregistered and subtracted images, of 7 DMF-treated patients who presented with LMCE foci at baseline, 5 continued to show the same LMCE foci at 12 and 24 months, whereas one new patient treated with DMF developed a new LMCE focus over the follow-up. One DMF-treated patient that showed one LMCE foci at post-contrast 3D_FLAIR did not obtain pre/post-contrast 3D-FLAIR protocol. Of 4 TFM-treated patients who presented with LMCE foci at baseline, 2 patients continued to present the same LMCE foci at 12 and 24 months, whereas one patient treated with TFM developed a new LMCE focus over the follow-up.

### 3.5. Differences in the MRI Lesion Activity Measures between the Two Treatment Groups

Table 5 shows no significant differences in the MRI lesion number or volumes between the two treatment groups over the follow-up.

## 4. Discussion

This was a retrospective, real-world, longitudinal study comparing the effectiveness of TFM and DMF on slowing cortical pathology in patients with relapsing MS, initiating treatment in clinical practice. The main study finding is that TFM showed superior effects on preservation of cortical GM volume, compared to DMF. We observed also a significant effect of TFM on slowing GM volume loss over 12 and 24 months, and development of central atrophy over 12 months of the study, compared to DMF. The TFM group also showed a numerically lower rate of whole brain volume loss over 24 months, compared to DMF. No differences in the frequency and number of LMCE foci were observed between the two treatment groups over the follow-up. Clinical and MRI lesion outcomes of disease activity were comparable between the two treatment groups over 24 months.

While it is difficult to detect cortical subpial lesions in vivo, the extent of cortical pathology in patients with MS can be indirectly assessed by measuring cortical atrophy [2,4,6].

Evidence is mounting that TFM can slow down cortical atrophy from the earliest clinical stages. In the TOPIC study [20], TFM reduced significantly cortical GM volume loss versus placebo over 24 months in CIS patients [37]. The results from the present study support these findings, as we found that TFM reduced the rate of cortical GM atrophy, compared to DMF. The effect size for PCVC between TFM and DMF in the current study (*d* = 0.66) was somewhat higher than the one recently observed for PBVC (*d* = 0.53) in the Teri-RADAR study [28]. These findings are also supported by a significant reduction of GM volume loss over 12 and 24 months in the TFM- compared to the DMF-treatment group. However, we did not detect differences between TFM and DMF treatment in the rate of thalamic and DGM atrophy in the whole cohort, although there were significant differences between TFM and DMF patients who were scanned only on the 3T MRI. It has been demonstrated previously that the humoral immune response to Epstein–Barr virus (EBV) is associated with more advanced development of cortical atrophy in MS patients [38]. Therefore, it could be hypothesized that a reduction of the humoral response to EBV by DMTs could contribute to slowing the development of cortical atrophy in MS patients. In a recent study, TFM-treated MS patients showed a similar rate of cortical atrophy over 12 months versus age- and sex-matched healthy controls [13], an effect that could have been mediated by the alteration of the humoral response to EBV [39]. In fact, the TFM-treated patients who decreased the most in their humoral response to anti-EBV were those who developed the lowest decline in cortical volumes over 12 months [39]. These preliminary findings are also supported by another recent in vitro study showing that TFM inhibited cellular proliferation, and promoted apoptosis, in EBV-transformed B cells at a clinically-relevant dose, suggesting that TFM may inhibit lytic EBV infection both by preventing the initial steps of viral reactivation and by blocking viral DNA replication, via its impact on host pyrimidine metabolism [40]. The potential anti-viral effect of TFM on the development of cortical pathology needs to be explored further.

Given that cortical atrophy and LM inflammation are emerging from pathology and imaging studies as important pathological substrates for cognitive and physical disease progression in MS [2,4,6], we hypothesized that the decrease of LMCE frequency may be associated with the decline of cortical atrophy. To the best of our knowledge, this is one of the first head-to-head studies to evaluate the effect of DMTs on LM inflammation, as measured by LMCE. At baseline, 10.3% of TFM- and 16.7% of DMF-treated patients were positive for LMCE on post-contrast 3D-FLAIR evaluation, which is a similar frequency to what was recently reported in relapsing MS patients using subtraction imaging between pre- and post-contrast 3D-FLAIR on 3T MRI in a clinical routine [36], however lower than previously reported using only the post-contrast 3D-FLAIR protocol only [4]. The differences between the two studies may be due to the more benign nature of patients selected for the present study, with respect to the previous one [4]. Approximately 75% of the subjects included in this study, who were evaluated on 3T MRI for LMCE, had also pre/post-contrast 3D-FLAIR subtraction evaluation for presence of LMCE [36]. We did not find any discrepancy in LMCE identification between the two subsets of TFM and DMF patients who were scanned on 3T with post-contrast 3D-FLAIR only or with pre/post-contrast 3D-FLAIR protocols. Over the follow-up, two TFM- and two DMF-treated patients were found to no longer have LMCE foci, while another TFM- and another DMF-treated patient developed LMCE foci. The low frequency of LMCE changes observed in the present study is in line with a previous report [2]. The negligible degree of LMCE activity prevented us from exploring its association with the development of cortical atrophy. While our study was underpowered to detect LMCE differences between the two treatments groups, it questions the feasibility of LMCE foci detection for use in clinical trials and clinical routine monitoring of MS on 3T MRI.

In the Teri-RADAR study, annualized PBVC was significantly lower with TFM versus DMF treatment, suggesting a greater effect of TFM on slowing down whole brain atrophy [28]. In the present study, although we found a numerically lower rate of whole brain atrophy over 24 months, and a significant reduction in the development of central atrophy over the first 12 months in TFM-treated patients, we did not replicate Teri-RADAR findings; however, our results corroborate findings from the pivotal phase 3 trials [21,22] and real-world studies [28]. The higher rate of brain volume loss in the first 12 months, compared to the 24 months of treatment, was previously attributed to the changes in water shifts (pseudoatrophy effect), induced by the potent anti-inflammatory effect of DMTs [41]. For example, post-marketing observational studies corroborated that the higher loss of brain volume in natalizumab patients is probably due to the pseudoatrophy effect [42,43,44]. However, the apparent brain volume loss is mostly attributed to reduced WM, rather than GM, volume [44], which is exactly the opposite of what we found in the current study. In fact, we did not observe significant or numerical differences in the PBVC and PWMVC between TFM and DMF in the first 12 months of the study, findings that were corroborated by additional analysis performed only on the 3T subcohort. As age and disease duration advances in MS patients, the disease transitions towards a less inflammatory and more neurodegenerative course [45]. In addition, it was found in a recent meta-analysis study of 38 clinical trials that the efficacy of immunomodulatory DMTs on MS disability strongly decreased with advancing age [46], which would indicate that there is little anti-inflammatory benefit to receiving immunomodulatory DMTs in older MS patients. Given the relatively older age of the MS population selected in this retrospective study, it is important to understand whether there is additional acceleration of the rate of brain atrophy in the aging MS population. The annualized rate of whole brain atrophy in both TFM and DMF treatment arms was somewhat higher than previously reported [47,48], which would indicate that the part of brain volume loss in this study is also possibly due to an aging-related effect.

The present study was not powered to examine differences in clinical and MRI lesion activity outcomes. Nevertheless, we examined clinical and MRI lesion activity measures in a post-hoc analysis between the two treatment groups. In line with several recent comparative effectiveness real-world studies between TFM and DMF [28,49,50], but contrary to others [51,52], we did not find any significant clinical (relapse rate and change in EDSS score) or MRI (new/enlarging T2 lesions, T1 pre- and post-contrast lesions and their volumes) differences between the two treatment groups in the current study. There were no unexpected safety signals for either treatment and the discontinuation rate was similar between the two treatment arms, as reported recently [53].

A major limitation of the present study was that it is retrospective, non-randomized, observational, and only MRI-blinded, as opposed to a randomized, controlled clinical trial, which limits our results to only a particular selected group of MS patients. However, we made a particular effort in the patient selection, matching the two treatment groups for age, disease duration, and disability. Moreover, all patients in our center are routinely assessed with standardized clinical and MRI protocols on an annual basis, which allowed us to collect these outcomes in a retrospective fashion. Although one of the study aims was to explore the differences between the LMCE foci in the two treatment groups over the follow-up, the study was limited, as the LMCE was examined only on 3T 3D-FLAIR sequence, which was not available for all patients at baseline. Furthermore, due to the observed cessation over the course of the study, Type II error and non-random cessation may be an issue. However, subjects who stopped the treatment were similar to those who did not in baseline demographic, MRI, and clinical characteristics (data not shown). As such, we are confident the loss of subjects was at random. In addition, the patients were scanned on 1.5T and 3T scanners, which could have accounted for some bias; however, the post-hoc analysis on the 3T subcohort confirmed our main findings of the study. In addition, we did not investigate the cognitive outcomes between the two treatment groups and their association with brain volume changes, which should be explored in future studies. Another limitation is that possibly a longer follow-up is needed to determine clinical and MRI differences between TFM and DMF in the clinical routine. Finally, the cohort of TFM and DMF patients selected in this retrospective study was older compared to those reported in the pivotal trials [18,19,24,25], but similar to another recent multicenter study conducted in the United States [28], which may represent a somewhat different clinical routine reality of patients starting TFM treatment. Therefore, the brain volume changes between TFM and DMF observed in this study have to be confirmed in younger study populations.

In conclusion, these findings suggest that TFM has a superior effect on the preservation of cortical GM volume, compared to DMF. This study also suggests limited utility of LMCE assessment in the clinical routine monitoring of MS patients.

## Figures and Tables

**Table 1:** Demographic and clinical characteristics of MS patients treated with teriflunomide or dimethyl fumarate at baseline.

	TFM (*n* = 60)	DMF (*n* = 60)	*p*-Value
Age, mean (SD)	50.9 (8)	50.8 (7.5)	0.879
Age onset, mean (SD)	33.5 (10.2)	33.8 (9.7)	0.869
Disease duration, mean (SD)	16.3 (8.6)	15.6 (9.6)	0.674
Sex, *n* (%)			0.820
Female	47 (78.3)	49 (81.7)	
Male	13 (21.7)	11 (18.3)	
BMI, mean (SD)	27.2 (5.9)	28.8 (6.1)	0.171
Race, *n* (%)			0.606
Caucasian	45 (75)	47 (78.3)	
African-American	7 (11.7)	10 (16.7)	
Hispanic	8 (13.3)	3 (5)	
EDSS at baseline, median (IQR)	3.0 (2.5–3.5)	2.75 (2.0–3.5)	0.377
Relapse rate in previous 12 months, mean (SD)	0.28 (0.49)	0.38 (0.66)	0.351
Relapse rate in previous 24 months, mean (SD)	0.38 (0.66)	0.35 (0.63)	0.779
DMT previous 12 months, *n* (%)			0.144
Interferon-beta 1a	18 (30)	23 (38.3)	
Glatiramer acetate	9 (15)	9 (15)	
Natalizumab	15 (25)	11 (18.3)	
Oral	4 (6.7)	0 (0)	
Other	0 (0)	3 (5)	
No treatment	14 (23.3)	14 (23.3)	

MS-multiple sclerosis; TFM-teriflunomide; DMF-dimethyl fumarate; BMI-body mass index; EDSS-Expanded Disability Status Scale; IQR-interquartile range; DMT-disease-modifying therapy. Oral treatment of selected patients treated previously with fingolimod. Other medications included intravenous immunoglobulin (*n* = 2) and mitoxantrone (*n* = 2). *p*-values derived from chi-squared test, Student’s *t*-test, and Mann–Whitney rank-sum test.

**Table 2:** Baseline MRI characteristics of MS patients treated with teriflunomide or dimethyl fumarate at baseline.

Lesion Measures	TFM (*n* = 60)	DMF (*n* = 60)	*p*-Value
T2 LN, mean (SD)	25.5 (15.6)	23 (15.3)	0.393
T2 LV, mean (SD)	15.3 (14.9)	12.5 (9.7)	0.226
T1 LN, mean (SD)	12.1 (10.9)	11.2 (8.9)	0.647
T1 LV, mean (SD)	4.6 (6.3)	2.8 (3.3)	0.062
Gd positivity, *n* (%)	10 (16.7)	13 (21.7)	0.626
Gd LN, mean (SD)	0.3 (1.4)	0.3 (0.8)	0.979
Gd LV, mean (SD)	0.2 (0.08)	0.05 (0.14)	0.230
LMCE *			
Positivity, *n* (%)	4 (10.3)	8 (16.7)	0.389
Number, mean (SD) sum	0.07 (0.3) 4	0.14 (0.3) 8	0.718
LMCE **			
Positivity, *n* (%)	4 (13.8)	7 (19.4)	0.742
Number, mean (SD) sum	0.06 (0.3) 4	0.1 (0.3) 7	0.684
**Brain Volume Measures**			
BPV, mean (SD)	1480.3 (91.9)	1476.3 (83)	0.804
GMV, mean (SD)	724.5 (66.9)	725.8 (50)	0.907
WMV, mean (SD)	755.8 (52.8)	750.5 (48.3)	0.572
LVV, mean (SD)	48.8 (18.1)	48.3 (19.8)	0.842
CV, mean (SD)	573.5 (56.6)	578.7 (41)	0.573
Thalamus volume, mean (SD)	19 (2.6)	18.7 (2.4)	0.432
DGM volume, mean (SD)	55.9 (6.6)	55.5 (6)	0.748

MS-multiple sclerosis; TFM-teriflunomide; DMF-dimethyl fumarate; LN-lesion number; LV-lesion volume; Gd-gadolinium; LMCE-leptomeningeal contrast enhancement; BPV-brain parenchymal volume; GMV-gray matter volume; WMV-white matter volume; LVV-lateral ventricle volume; CV-cortical volume; DGM-deep GM volume. * LMCE was evaluated only on post-contrast 3D-FLAIR sequence on a 3T scanner, which was present in 39 TFM and 48 DMF patients at baseline. ** LMCE was evaluated on pre/post-contrast coregistered and subtracted 3D-FLAIR images on a 3T scanner, which was present in 29 TFM- and 36 DMF-treated patients at baseline, Volumes are in milliliters. Brain volumes are normalized for head size. *p*-values derived from ANCOVA, corrected for age and sex.

**Table 3:** Clinical characteristics of MS patients treated with teriflunomide or dimethyl fumarate over the follow-up.

	TFM (*n* = 48) *	DMF (*n* = 46) *	*p*-Value
Cessation of original DMT			0.214
0–12 months, *n* (%)	0 (0)	3 (5)	
12–24 months, *n* (%)	12 (20)	11 (18.3)	
EDSS at 12 months, median (IQR)	3.5 (2.5–4.0)	3.0 (2.0–4.0)	0.377
EDSS at 24 months, median (IQR)	3.5 (3.0–4.4)	3.0 (2.0–4.0)	0.339
EDSS absolute change between 0–12 months, mean (SD)	0.1 (1)	0 (0.7)	0.342
EDSS absolute change between 12–24 months, mean (SD)	0.1 (0.9)	0.2 (0.5)	0.626
EDSS absolute change between 0–24 months, mean (SD)	0.2 (1)	0.2 (0.8)	0.722
Relapse rate between 0–12 months, mean (SD) sum	0.25 (0.4) 13	0.21 (0.5) 13	0.683
Relapse rate between 12–24 months, mean (SD) sum	0.1 (0.3) 6	0.15 (0.4) 9	0.412
Annualized relapse rate between 0–24 months, mean (SD) sum	0.17 (0.4) 19	0.18 (0.5) 22	0.890
DMT after cessation of original treatment, *n* (%)			0.950
Interferon beta-1a	7 (11.7)	7 (11.7)	
Glatiramer acetate	3 (5)	3 (5)	
Natalizumab	2 (3.3)	4 (12.5)	

Note: MS-multiple sclerosis; TFM-teriflunomide; DMF-dimethyl fumarate; BMI-body mass index; EDSS-Expanded Disability Status Scale; IQR-interquartile range; DMT-disease-modifying therapy. * At 12 months, 60 TFM- and 57 DMF-treated patients completed the follow-up, whereas at 24 months 48 TFM- and 46 DMF-treated patients completed the follow-up. *p*-values derived from chi-squared test, Student’s *t*-test, and Mann–Whitney rank-sum test.

**Table 4:** MRI brain volume characteristics of MS patients treated with teriflunomide or dimethyl fumarate over the follow-up.

	0–12 Months			12–24 Months			0–24 Months		
	TFM (*n* = 60)	DMF (*n* = 57)	*p*-Value	TFM (*n* = 48)	DMF (*n* = 46)	*p*-Value	TFM (*n* = 48)	DMF (*n* = 46)	*p*-Value
PBVC	−0.73 (1.4)	−0.92 (1.5)	0.496	−1.08 (1.2)	−1.4 (1.7)	0.351	−1.45 (1.5)	−2.07 (1.7)	*0.077*
PGMVC	−0.24 (3.9)	−1.71 (3)	**0.044**	−075 (5.1)	−1.33 (4.1)	0.605	−0.44 (5.2)	−3.12 (4.3)	**0.015**
PWMVC	−1.72 (4.7)	−1.19 (4.1)	0.565	−2.87 (5.1)	−2.24 (5.1)	0.618	−4.88 (4.8)	−4.17 (5.1)	0.550
PLVVC	1.40 (5.2)	3.56 (5.7)	**0.05**	3.07 (7.5)	2.25 (6.9)	0.620	3.85 (10.2)	6.26 (8.7)	0.245
PCVC	−0.29 (4)	−1.48 (3.2)	0.115	−0.79 (3.9)	−1.6 (3.3)	0.358	−0.20 (4.3)	−2.94 (3.9)	**0.004**
PTVC	−0.83 (4)	−1.20 (3.3)	0.624	−0.44 (5)	−1.1 (4.1)	0.531	−1.18 (5.5)	−1.87 (5)	0.556
PDGMVC	−0.5 (3.8)	−0.91 (3.2)	0.557	−0.34 (5.4)	−1.03 (4.4)	0.538	−1.0 (5.6)	−1.7 (4.2)	0.515

Note: TFM-teriflunomide; DMF-dimethyl fumarate; PBVC-percentage brain volume change; PGMVC-percentage gray matter volume change; PWMVC-percentage white matter volume change; PLVVC-percentage lateral ventricle volume change; PCVC-percentage cortical volume change; PTVC-percentage thalamus volume change; PDGMVC-percentage deep GM volume change. *p*-values derived from ANCOVA, corrected for age and sex. *p*-values shown in bold and italics are ≤0.05 and ≤0.1, respectively.

**Table 5:** MRI lesion characteristics of MS patients treated with teriflunomide or dimethyl fumarate over the follow-up.

	0–12 Months			12–24 Months			0–24 Months		
	TFM (*n* = 60)	DMF (*n* = 57)	*p*-Value	TFM (*n* = 48)	DMF (*n* = 46)	*p*-Value	TFM (*n* = 48)	DMF (*n* = 46)	*p*-Value
No. of new/enlarging T2 lesions, mean (SD)	0.1 (0.5)	0.1 (0.2)	0.121	0.1 (0.2)	0.1 (0.4)	0.367	0.2 (0.6)	0.2 (0.6)	0.945
Change in absolute T2-LV, mean (SD)	0.3 (1.5)	0.2 (0.8)	0.620	−0.4 (1.9)	−0.2 (1.3)	0.598	−0.1 (2.4)	−0.1 (1.5)	0.990
No. of new/enlarging T1 lesions, mean (SD)	0.1 (0.4)	0.06 (0.3)	0.309	0.06 (0.2)	0.08 (0.3)	0.720	0.23 (0.6)	0.18 (0.5)	0.593
Change in absolute T1-LV, mean (SD)	0.04 (0.5)	0.005 (0.3)	0.169	0.08 (0.6)	0.1 (0.7)	0.175	0.1 (0.7)	0.13 (0.7)	0.821
Gd positivity, *n* (%)	3 (5)	2 (3.3)	0.690	2 (4.2)	3 (6.5)	0.611	3 (6.3)	4 (8.7)	0.651
Change in Gd-LV, mean (SD)	0.06 (0.3)	−0.04 (0.2)	0.151	−0.06 (0.7)	0.06 (0.3)	0.205	−0.01 (0.1)	−0.01 (0.1)	0.495
LMCE positivity *, *n* (%)	2 (5.7)	6 (12.8)	0.157	3 (10)	5 (12.5)	0.998	3 (10)	7 (17.5)	0.375
LMCE No. *, mean (SD)	0.04 (0.2)	0.1 (0.3)	0.109	0.07 (0.3)	0.1 (0.3)	0.424	0.1 (0.5)	0.2 (0.7)	0.216
LMCE positivity **, *n* (%)	2 (6.9)	5 (13.9)	0.140	3 (13)	5 (16.7)	0.991	3 (13)	6 (20)	0.715
LMCE No. **, mean (SD)	0.04 (0.1)	0.08 (0.2)	0.211	0.09 (0.3)	0.1 (0.2)	0.375	0.09 (0.4)	0.2 (0.6)	0.199

Note: TFM-teriflunomide; DMF-dimethyl fumarate; LV-lesion volume; Gd-gadolinium; LMCE-leptomeningeal contrast enhancement. * LMCE was evaluated only on post-contrast 3D-FLAIR sequence on a 3T scanner, which was present in 39 TFM- and 48 DMF-treated patients at baseline, 35 TFM- and 47 DMF-treated patients at 12 months, and 30 TFM- and 40 DMF-treated patients at 24 months. ** LMCE was evaluated on pre/post-contrast coregistered and subtracted 3D-FLAIR images on a 3T scanner, which was present in 29 TFM- and 36 DMF-treated patients at baseline, 26 TFM- and 35 DMF-treated patients at 12 months, and 23 TFM- and 30 DMF-treated patients at 24 months. Volumes are in milliliters. *p*-values derived from chi-squared test and ANCOVA, corrected for age and sex.

## Supplementary Materials

The following are available online at https://www.mdpi.com/2077-0383/8/3/344/s1, Table S1: MRI brain volume characteristics of MS patients treated with teriflunomide or dimethyl fumarate over the follow-up, who were examined only on the 3T scanner.
