# Supplementary material for: Effect of Teriflunomide and Dimethyl Fumarate on Cortical Atrophy and Leptomeningeal Inflammation in Multiple Sclerosis: A Retrospective, Observational, Case-Control Pilot Study
Source: J Clin Med. 2019 Mar 12;8(3):344. doi: 10.3390/jcm8030344 (PMC6463015; doi:10.3390/jcm8030344)
Supplement: Supplementary file 1 [file jcm-08-00344-s001.pdf]

Table S1. MRI brain volume characteristics of MS patients treated with teriflunomide or dimethyl fumarate over the follow-up, who were examined only on the 3T scanner.

	**0–12 Months**			**12–24 Months**			**0–24 Months**		
	**TFM (*n* = 39)**	**DMF (*n* = 45)**	***p*-Value**	**TFM (*n* = 31)**	**DMF (*n* = 37)**	***p*-Value**	**TFM (*n* = 31)**	**DMF (*n* = 37)**	***p*-Value**
PBVC	−0.98 (1.4)	−1.1 (1.5)	0.876	−1.04 (1.1)	−1.30 (1.7)	0.530	−1.50 (1.6)	−2.1 (1.6)	0.188
PGMVC	−0.34 (4.1)	−1.81 (2.9)	*0.086*	−0.54 (4.1)	−1.69 (4.1)	0.374	−0.57 (4.7)	−3.4 (4.4)	**0.001**
PWMVC	−2.07 (4.3)	−1.60 (4.1)	0.288	−2.69 (5)	−1.9 (3.5)	0.324	−4.53 (4.5)	−3.90 (4.7)	0.347
PLVVC	0.2 (5.1)	2.2 (5)	0.324	1.8 (3.7)	1.7 (3.9)	0.970	2.6 (4.5)	4.2 (6.2)	0.165
PCVC	-0.87 (3.8)	−1.86 (2.7)	0.183	−0.49 (4)	−1.65 (3.2)	0.254	−0.35 (4)	−3.34 (3.9)	**0.01**
PTVC	−1.14 (2.3)	−3.1 (2.7)	0.225	−0.61 (3.2)	−2.26 (4.5)	*0.058*	−1.43 (3.5)	−3.69 (5.8)	**0.046**
PDGMVC	−0.6 (3.9)	−1.8 (3.4)	0.385	−0.68 (4.5)	−1.5 (3.4)	**0.037**	−1.34 (4.3)	-4.1 (5.6)	**0.008**

TFM-teriflunomide; DMF-dimethyl fumarate; PBVC-percentage brain volume change; PGMVC-percentage gray matter volume change; PWMVC-percentage white matter volume change; PLVVC-percentage lateral ventricle volume change, PCVC-percentage cortical volume change; PTVC-percentage thalamus volume change; PDGMVC-percentage deep GM volume change. *p*-values derived from ANCOVA, corrected for age and sex. *p*-values shown in bold and italics are ≤0.05 and ≤0.1, respectively.
